# Hybrid Antimicrobial Peptide Targeting *Staphylococcus aureu*s and Displaying Anti-infective Activity in a Murine Model

**DOI:** 10.3389/fmicb.2020.01767

**Published:** 2020-09-11

**Authors:** Lu Shang, Jiawei Li, Chunsheng Song, Zaytseva Nina, Qiuke Li, Shuli Chou, Zhihua Wang, Anshan Shan

**Affiliations:** Institute of Animal Nutrition, Northeast Agricultural University, Harbin, China

**Keywords:** antibiotic resistance, *S. aureus*, antimicrobial peptides, targeting antimicrobial activity, subcutaneous infection

## Abstract

Broad-spectrum antimicrobial peptides (AMPs) kill bacteria indiscriminately, increasing the possibility of an ecological imbalance in the microbiota. To solve this problem, new types of AMPs, which kill pathogenic bacteria without breaking the micro-ecological balance of the body, were proposed. Here, we successfully designed a targeting AMP, S2, which is a fusion peptide composed of a species-specific targeting domain and broad-spectrum AMP domain. In the current study, S2 showed specific killing activity against *Staphylococcus aureus*, and almost no resistance induced compared to penicillin. Mechanism studies indicated that S2 killed *S. aureus* by destroying the bacterial membrane. Meanwhile, S2 possessed excellent salt-tolerance properties and biocompatibility. Importantly, S2 exhibited perfect treatment efficacy against an *S. aureus* subcutaneous infection model and remained nontoxic. In conclusion, this study provides a promising strategy for designing specific AMPs against growing bacterial infections.

## Introduction

Methicillin-resistant *Staphylococcus aureus* (MRSA) is one of the primary pathogens of global nosocomial and community-associated infections and is responsible for diseases ranging from localized skin infections to severe sepsis, including bacteremia, endocarditis, and toxic shock syndrome (Krut et al., 2006; Park et al., 2008; Rollin et al., 2017). Vancomycin has long been considered the drug of choice for the treatment of infections caused by MRSA. However, the emergence of vancomycin-resistant *Staphylococcus aureus* (VRSA) and vancomycin-intermediate *Staphylococcus aureus* (VISA) strains pose a serious threat to the treatment of MRSA infections (Perichon and Courvalin, 2009; Assis et al., 2017; Cao et al., 2018). Although many new antibacterial drugs have been found to cure the infections of *S. aureus* clinically, their antimicrobial mechanisms are similar to those of conventional antibiotics, thus raising concerns about the continued trend of drug-resistant bacteria (Huang et al., 2011a). Therefore, we need to find an effective, durable antibiotic substitute as soon as possible.

Naturally occurring antimicrobial peptides (AMPs) with positive charges and hydrophobic amino acids are the endogenous defense molecules of living organisms (Zarena et al., 2017). In emerging multidrug-resistant bacteria, AMPs not only kill bacteria, fungi, viruses, and parasites but also display wound healing, endotoxin neutralization, anti-biofilm, and septic shock relief properties (Wang et al., 2019a). Thus, AMPs show great potential to replace traditional antibiotics for curing difficult-to-treat infections. Moreover, contrary to conventional antibiotics that target certain biosynthetic pathways crucial to cell wall or protein synthesis, the majority of AMPs disrupt the membrane and cause rapid membrane permeabilization through physical means, which effectively kills multidrug-resistant bacteria (Ma et al., 2016; Wang et al., 2019a). However, broad-spectrum AMPs may result in the selection and proliferation of resistant bystander microorganisms, creating pathogens from previously harmless organisms and causing complications such as antibiotic-associated diarrhea and colitis (Qiu et al., 2003).

The *agr* system of *staphylococci* is a quorum sensing system that controls the expression of exoproteins and surface proteins (Block et al., 2017; Choudhary et al., 2018). The resulting mature autoinducing peptide (AIP) consists of 7–9 amino acid residues with a five-residue thiolactone macrocycle (Karathanasi et al., 2018). A previous study has shown that the pheromone of *S. aureus* fused to plectasin can selectively kill *S. aureus* (Xi et al., 2013). However, the thiolactone macrocycle in the pheromone of *staphylococci* is expensive and difficult to synthesize. Therefore, we synthesized a parent peptide (CASYFCRWWWLL-NH_2_) by removing the thiolactone macrocycle of the pheromone and linking it to the short AMP. Based on the above, we further investigated whether adding disulfide bonds to the targeting domain can affect the antimicrobial capacity of the peptide. The targeting antimicrobial activity of AMPs not only depends on the targeting domain, but also on the killing domain. Thus, we designed a series of killing domains against *S. aureus* for the following reasons: (1) Tryptophan (W) has the unique ability to interact with membranes and facilitate peptide anchoring to bacterial surfaces, and the π configuration of the WWW motif of the short tryptophan-rich peptide is critical for the targeting killing of MRSA (Zarena et al., 2017); (2) Hydrophobicity is strongly correlated with antimicrobial activity and cell selectivity. AMPs with the appropriate hydrophobicity can damage the membrane structure, causing cell lysis (Yang et al., 2019); (3). Since the negatively charged components are the first line of the cell membrane that AMPs interact with, the effects of the positive charge on the antimicrobial potency are evident (Lyu et al., 2019); and (4). The killing domain of the hybrid peptide affects structural parameters, resulting in different antimicrobial potencies (Eckert et al., 2006; Ma et al., 2015).

**SCHEME 1:**
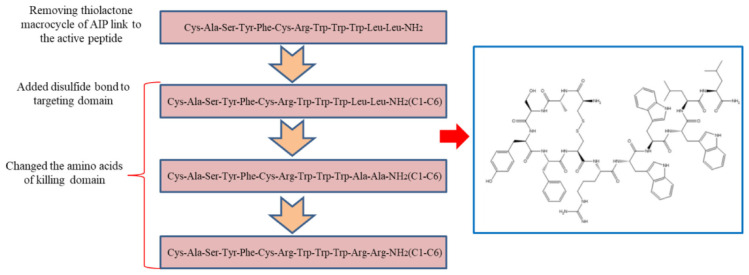
General schematic of designed peptides.

In this study, we used a combination of multiple strategies and modified the AIP to design AMP that target *S. aureus* (Scheme 1). The antimicrobial activities of peptides against different Gram-negative and Gram-positive microbes were determined by minimum inhibitory concentration (MIC) assays. The mechanism of action was illustrated by fluorescence spectroscopy and electron microscopy. In addition, the salt tolerance of peptides was tested in the presence of various cations at physiological concentrations and the hemolytic activity and cytotoxicity of peptides were observed. Eventually, a mouse model was used to determine the *in vivo* efficiency of S2.

## Materials and Methods

### Bacterial Strains

The bacterial strains *Escherichia coli* (*E. coli*) ATCC 25922, *E. coli* K88, *E. coli* K99, *Pseudomonas aeruginosa* (*P. aeruginosa*) ATCC 27853, *Escherichia coli* (*E. coli*) UB1005, *Salmonella typhimurium* 14028, *Salmonella pullorum* (*S. pullorum*) C7913, *S. typhimurium* C7731, *S. aureus* ATCC 29213, MRSA ATCC 43300, and *S. aureus* ATCC 25923 were obtained from the College of Veterinary Medicine, Northeast Agricultural University, China. *Lactobacillus plantarum* (*L. plantarum*) 8014, *Lactobacillus rhamnosus* (*L. rhamnosus*) 7469, *L. rhamnosus* 1.0911, *L. rhamnosus* 1.0936, and *L. rhamnosus* 1.0385 were obtained from the Key Laboratory of Food College, Northeast Agricultural University, China.

### Materials

Mueller-Hinton broth (MHB), Muller-Hinton Agar (MHA), and MRS media were purchased from AoBoX (Shanghai, China). Human red blood cells (hRBCs) were donated from volunteers with healthy blood. Analytical-grade reagents (sodium chloride, potassium chloride, ammonium chloride, zinc chloride, magnesium chloride, and ferric chloride) were purchased from Kermel (China). Sodium dodecyl sulfate (SDS) micelles, ethanol (analytical grade, 99%), tertiary butanol (analytical grade, 99%), glutaraldehyde (synthetic grade, 50% in H_2_O), BODIPY-TR-cadaverine (BC), lipoteichoic acid (LTA) from *S. aureus*, Triton X-100, 3,3′-dipropylthiadicarbocyanine iodide (diSC_3_-5), 3-(4,5-dimethylthiazol-2-yl)-2,5-diphenyltetrazolium bromide (MTT), propidium iodide (PI), and HEPES were purchased from Sigma-Aldrich (China). RPMI-1640 medium supplemented with 10% fetal calf serum and 1% penicillin and streptomycin mixture was obtained from Invitrogen Corporation (United States).

### Peptide Synthesis and Analysis

All peptides were synthesized by Sangon Biotech (Shanghai, China). The purity of the peptides was >95% [confirmed by analytical reverse-phase high-performance liquid chromatography (RP-HPLC)]. The fidelity of the peptides was determined via matrix-assisted laser desorption/ionization time-of-flight mass spectrometry (MALDI-TOF MS, Linear Scientific Inc., United States), and α-cyano-4-hydroxycinnamic acid was used as the matrix.

### Antimicrobial Assays

As in a previous study, the antimicrobial activities of the AMPs were investigated using a modified standard microtiter dilution method (Dong et al., 2014). The peptides were serially diluted with a solution containing 0.01% acetic acid and 0.2% bovine serum albumin (BSA, Sigma) in 96-well plates. The bacteria grew to the mid-log phase and then were dissolved in MHB at concentration of 2–7 × 10^5^ CFU/ml. The peptides and bacterial solution were mixed in equal volume in a 37°C incubator for 24 h. The lowest concentration that showed no turbidity was recorded as the MIC value. Mueller-Hinton broth with microorganisms was used as the positive control, and the pure broth was used as the negative control. The experiment was repeated at least three times independently.

### Time-Dependent Killing Kinetics

The logarithmic phase *S. aureus* ATCC 29213 cells were harvested by centrifugation at 1000 × g for 5 min, washed twice with PBS, and diluted to 2–7 × 10^5^ CFU/mL. *S. aureus* was treated with AMPs at various concentrations, including 0.125, 0.25, 0.5, 1, and 2 × MIC. At various time intervals (5 s, 10 s, 30 s, 1 min, 5 min, 10 min, and 30 min), 50 μL of bacterial solution was diluted and plated on MHA plates. Bacterial colonies were counted after 24 h of incubation at 37°C. The results were carried out in six independent trials.

### Evaluation of Bacterial Resistance

*Staphylococcus aureus* 29213 was employed to assess the development of resistance by the serial passaging method, as previously described (Lyu et al., 2017). Minimum inhibitory concentration testing was first conducted for all of the compounds to be tested, as described above. *S. aureus* was harvested from 96-well plates of sub-MIC levels, diluted in MHB to a concentration of 2–7 × 10^5^ CFU/mL, and then subjected to MIC determination for up to 15 days. The change in MIC values determines the development of antibiotic resistance in *S. aureus*.

### Circular Dichroism Analysis

Circular dichroism spectra were obtained on a J-820 spectropolarimeter (Jasco, Tokyo, Japan). Measurements were carried out at 25°C using a 0.1 cm path length rectangular quartz cell. The peptides were analyzed in 10 mM PBS (pH 7.4), 50% trifluoroethyl alcohol (TFE) to mimic the hydrophobic environment of the microbial membrane, and 30 mM SDS micelles (Wang et al., 2018). Samples were scanned from 195 to 250 nm at a speed of 10 nm/min. The final concentration of peptides was 150 μM. The mean residue ellipticity was calculated to illustrate the CD spectra using the following equation: θ_*M*_ = (θobs × 1000)/(c × l × n). where θ_*M*_ represents the mean residue ellipticity (deg cm^2^/dmol); θ_*obs*_ represents the observed ellipticity, which was corrected for the buffer at a specific wavelength (mdeg); c represents the peptide concentration (mM); l represents the path length (mm); and n represents the number of amino acids (Dong et al., 2019).

### Peptide Interactions With LTA

Peptides binding to LTA were assayed using the BC (Sigma, United States) displacement method (Zorko and Jerala, 2008). The final concentration of 50 μg/mL LTA from *S. aureus* was incubated with 5 μg/mL BC in Tris-HCl buffer (50 mM, pH 7.4) for 4 h in a shaking incubator at 37°C. The peptides were then serially diluted in Tris–HCl buffer, and an equal volume solution was added to the 96-well plates. The fluorescence was measured (excitation λ = 580 nm, emission λ = 620 nm) on a spectrofluorophotometer (Infinite 200 Pro, Tecan, China). The experiment was conducted three times independently.

### Electrical Potential Measurement of Cytoplasmic Membrane

The dye diSC_3_-5 was employed to assay cytoplasmic membrane depolarization activities (Shao et al., 2018). *S. aureus* 29213 was diluted to an OD_600_ = 0.05 after washing three times with buffer (5 mM HEPES, 5 mM glucose, pH 7.4) when the bacteria grew to mid-log phase. Then, diSC_3_-5 at a final concentration of 0.4 μM was added into the medium and incubated for 1–1.5 h. The final concentration of 100 mM KCl was then added to the solution for 15 min. The peptides were added to 12-well plates, which were filled with 2 mL of cell suspension. An F-4500 fluorescence spectrophotometer (Hitachi, Japan) was employed to measure the fluorescence value at an excitation wavelength of 622 nm and an emission wavelength of 670 nm. Maximal augmentation was monitored, and the fluorescence of the mixture of bacteria and dye served as the blank control.

### SEM and TEM Characterization

Bacterial membrane damage induced by the peptide was visualized using scanning electron microscopy (SEM). *S. aureus* ATCC 29213 was washed three times using PBS after growing to the mid-log phase and diluted to an OD_600_ = 0.2. The bacteria were incubated for 30 min with peptide, and the bacterial growth in PBS was used as the control. The cells were washed twice with PBS and fixed with 2.5% (w/v) glutaraldehyde at 4°C overnight. The bacterial cells were dehydrated with a graded series of ethanol (50, 70, 90, and 100%) for 10 min at each step. The samples were transferred to a mixture (v: v = 1:1) of alcohol and tert-butanol for 30 min and tert-butanol alone for 1 h. The dried bacterial specimens, which were obtained using a critical point dryer, were coated and visualized under a field emission scanning electron microscope (Hitachi S-4800, Japan).

The processing method of the samples for transmission electron microscopy (TEM) was the same as the treatment for SEM. The samples were then prefixed with 2.5% glutaraldehyde at 4°C overnight, post-fixed with osmium tetroxide for 70 min, and washed twice with PBS (pH 7.2). Subsequently, the samples were dehydrated in different concentrations of ethanol (50, 70, and 90%) for 8 min in series and 10 min in 100% ethanol twice and then transferred to a mixture (v:v = 1:1) of 100% ethanol and acetone for 10 min. The samples were further embedded in a 1:1 mixture of absolute acetone and epoxy resin for 30 min and absolute epoxy resin overnight. Finally, the specimens were sectioned with an ultramicrotome, stained with uranyl acetate and lead citrate, and observed using a Hitachi H-7650 TEM(Wang et al., 2018).

### Propidium Iodide Uptake Assay

*Staphylococcus aureus* 29213 was washed three times with buffer (5 mM HEPES, 5 mM glucose, pH 7.4) after the bacterium grew to the mid-log phase and then diluted to an OD_600_ = 0.05. Propidium iodide was then added to the final concentration of 10 μg/mL and incubated for 30 min at 4°C. The peptides were added to 12-well plates, which were filled with 2 mL of the cell suspension. An F-4500 fluorescence spectrophotometer (Hitachi, Japan) was employed to measure the fluorescence value at an excitation wavelength of 535 nm and an emission wavelength of 617 nm. Maximal augmentation was monitored, and the fluorescence of the mixture of bacteria and dye served as the blank control (Morita et al., 2015).

### Cytotoxicity Measurements

The cytotoxicity of peptides was determined by healthy human erythrocytes and the murine macrophage cell line RAW 264.7 via a hemolysis assay and MTT dye reduction assay, respectively (Wang et al., 2018). In the hemolysis assay, fresh hRBCs from healthy people were centrifuged for 5 min at 1,000 × *g* and then washed three times with PBS (pH = 7.2). The 100 μL erythrocyte suspension and peptides were incubated for 1.5 h in an incubator at 37°C. The 96-well plates were centrifuged at 1,000 × *g* for 5 min, and the supernatants were carefully aspirated. The absorbance at 570 nm indicated the leakage of hemoglobin. The hRBCs in the PBS sample was used as the negative control, and 0.1% Triton X-100 addition served as the positive control. In the MTT dye reduction assay, 1.0–2.0 × 10^5^ cells were plated in 96-well plates and then treated with various concentrations of peptides for 4 h at 37°C in a 5% CO_2_ incubator. Subsequently, 50 μL of MTT was added to the plated cells at a final concentration of 0.5 mg/mL and incubated for 4 h. Then, the cells were centrifuged at 1000 × *g* for 5 min, and the supernatants were discarded. Formazan crystals were dissolved in 150 μL of DMSO, and the OD_570 *nm*_ was measured using a microplate reader (TECAN GENios F129004; TECAN, Austria). The experiment was repeated three times independently.

### Effect of Salts on Antimicrobial Activity

The MIC of peptides was determined in the presence of salts at physiological concentrations (150 mM NaCl, 4.5 mM KCl, 6 μM NH_4_Cl, 8 μM ZnCl_2_, 1 mM MgCl_2_, 2 mM CaCl_2_, and 4 μM FeCl_3_) as described previously (Dong et al., 2014). All experiments were performed three times independently.

### Antimicrobial Activity *in vivo*

To assess the antimicrobial activity of the peptide *in vivo*, a mouse model of subcutaneous infection was used (Huang et al., 2011a; Omolo et al., 2017). Female Institute for Cancer Research (ICR) mice (SPF, 20–25 g) were purchased from WeiTonglihua Co., Ltd. (Beijing, China), with eight animals per group. The mice were shaved on their backs and disinfected. *S. aureus* 29213 (100 μL) at a concentration of 1 × 10^8^ CFU/mL was injected into the superficial skin of the mice. The mice were divided into four groups (*n* = 4), as follows: *S. aureus* + saline, *S. aureus* + S2 (80× MIC, 100 μL), *S. aureus* + vancomycin (Van) (80× MIC, 100 μL), and saline. S2, vancomycin, and saline were injected at the same site 1 h after infection. The mice were monitored over 24 h in an environmentally controlled room at 25 ± 1°C and 55 ± 5% relative humidity with a 12 h light/dark cycle.

To image the infected skin tissues, the mice were euthanized 24 h after the injection of *S. aureus*. Samples were fixed in 4% buffered paraformaldehyde and subjected to hematoxylin/eosin (HE) staining. The antimicrobial activity *in vivo* was determined by colony counting of infected skin samples. Skin homogenates were serially diluted in saline and incubated at 37°C for 24 h.

### Cytokine Assay

The mice were treated as described above. Skin samples were treated according to the manufacturer’s instructions. The levels of tumor necrosis factor-alpha (TNF-α), interleukin-6 (IL-6), and granulocyte-macrophage colony-stimulating factor (GM-CSF) in infected skin were measured using enzyme-linked immunosorbent assay (ELISA) kits following the manufacturer’s instructions.

### Toxicity of Peptides *in vivo*

Anesthetized mice were shaved on their backs and disinfected. A total of 100 μL of S2 (80 × MIC) was injected into the skin. Equal volumes of saline and 100% DMSO were used as the negative and positive controls, respectively. The skin was removed from the injection sites for tissue processing after 24 h. OCT-embedded slides were stained using the DeadEnd^TM^ Fluorometric TUNEL System for the detection of apoptotic cells and counterstained with 4,6-diamidino-2-phenylindole (DAPI). Photographs were taken using a microscope (Huang et al., 2011a).

The mice were inoculated by intraperitoneal injection with 100 μL of S2 (80 MIC), and saline was used as the control. Signs of animal distress and changes in body weight were closely monitored. After five days, all the mice were euthanized, and the blood biochemistry parameters were determined (Mishra et al., 2019).

### Statistical Analysis

Statistical significance of the experimental results was determined by one-way ANOVA, followed by Duncan’s test. Differences with a value of *P* < 0.05 were considered statistically significant. Data were analyzed by the unpaired Student’s *t*-test or one-way ANOVA using SPSS 20.0 software (IBM, Chicago, IL, United States).

### Ethics Statement

This study was approved by the Animal Care and Use Committee of the Northeast Agricultural University, China. The animals used in this experiment were cared for under the guidelines stated in the Guide for the Care and Use of Agricultural Animals in Agricultural Research and Teaching of Heilongjiang Province, China. The measurement of hemolytic activity was reviewed and approved by the Ethics Committee of the Northeast Agricultural University Hospital, and the experimental method was carried out in accordance with the approved guidelines and regulations.

## Results

### Designed and Characterization of the Peptide

We designed and synthesized eight peptides that were 6–12 amino acids in length. The sequence and the net amounts of positive charge were shown in Table 1, and all peptides were amidated on the C-terminus. To screen the most effective AMP, we constructed a peptide library including S6, S7, and S8 by utilizing the structure-function relationship. Furthermore, S4 was synthesized to investigate the effect of the conjugation of the modified AIP removed thiolactone macrocycle to the killing domain on antimicrobial activity. S2 was designed based on S4 to determine whether the addition of a disulfide bond (Cys1–Cys6) to the targeting domain affected the antimicrobial spectrum. S1 and S3 were synthesized to determine the effects on the killing domain and S5 was synthesized to investigate whether the targeting domain possessed targeting killing activity. The theoretical molecular weights of all the peptides were consistent with the measured molecular weights, which indicated that all the peptides were synthesized successfully.

**TABLE 1:** Peptides design and key physicochemical parameters.

**Peptide**	**Sequence**	**TMW (Da)^a^**	**MMW (Da)^b^**	**Net charge**
S1	CASYFCRWWWRR-NH_2_ (C1–C6)	1717.03	1717	+4
S2	CASYFCRWWWLL-NH_2_ (C1–C6)	1630.98	1630.8	+2
S3	CASYFCRWWWAA-NH_2_ (C1–C6)	1546.82	1546.7	+2
S4	CASYFCRWWWLL-NH_2_	1630.98	1630.8	+2
S5	CASYFC (C1–C6)	689.83	689.3	+1
S6	RWWWRR-NH_2_	1044.23	1044	+4
S7	RWWWLL-NH_2_	958.18	958.1	+2
S8	RWWWAA-NH_2_	874.02	873.9	+2

*^*a*^TMW, theoretical molecular weight; ^*b*^MMW, measured molecular weight (measured by mass spectroscopy).*

### Antimicrobial Activity

To evaluate the antimicrobial activity and specificity of the peptides, MIC was tested against a panel of bacterial species. Tables 2, 3 show that S2 and S7 exhibited the same activity against *S. aureus*, with an MIC value of 8 μM. As expected, the addition of the targeting domain [CASYFC (C1–C6)] to S7 showed targeting antimicrobial activity against *S. aureus*. S3, S4, S5, and S8 failed to show antimicrobial activity against the tested microorganisms at the highest concentration measured. S6 and S7 showed poor activity against all tested bacteria. S1, at concentrations of 4–64 μM, suppressed the growth of all tested Gram-negative bacteria except for *P. aeruginosa* 27853. Importantly, all peptides in this study were inactive against probiotics, including *L. rhamnosus* and *L. plantarum*.

**TABLE 2:** The MICs of the peptides against gram-negative bacteria.

**Peptides/MIC (μM)^a^**	***E. coli* 25922**	***E. coli* K88**	***E. coli* K99**	***E. coli* UB1005**	***S. typhimurium* 14028**	***S. typhimurium* 7731**	***S. pullorum* 7913**	***P. aeruginosa* 27853**
S1	8	32	4	8	64	32	8	>64
S2	>64	>64	>64	>64	>64	>64	>64	>64
S3	>64	>64	>64	>64	>64	>64	>64	>64
S4	>64	>64	>64	>64	>64	>64	>64	>64
S5	>64	>64	>64	>64	>64	>64	>64	>64
S6	32	32	32	32	>64	>64	>64	>64
S7	16	16	16	16	32	16	>64	>64
S8	>64	>64	>64	>64	>64	>64	>64	>64

*^*a*^Minimum inhibitory concentration (MIC, μM) was determined as the lowest concentration of peptide with no microbial growth being observed.*

**TABLE 3:** The MICs of the peptides against gram-positive bacteria.

**Peptides/MIC (μM)**	***S. aureus* 29213**	***S. aureus* 25923**	**MRSA^a^ 43300**	***L. plantarum* 8014**	***L. rhamnosus* 7469**	***L. rhamnosus* 1.0911**	***L. rhamnosus* 1.0936**	***L. rhamnosus* 1.0385**
S1	4	4	4	>64	>64	>64	>64	>64
S2	8	8	8	>64	>64	>64	>64	>64
S3	>64	>64	>64	>64	>64	>64	>64	>64
S4	>64	>64	>64	>64	>64	>64	>64	>64
S5	>64	>64	>64	>64	>64	>64	>64	>64
S6	16	16	8	>64	>64	>64	>64	>64
S7	8	8	8	>64	>64	>64	>64	>64
S8	>64	>64	>64	>64	>64	>64	>64	>64

*^*a*^Methicillin-resistant *Staphylococcus aureus*.*

### Time-Dependent Killing Kinetics

**FIGURE 1:**
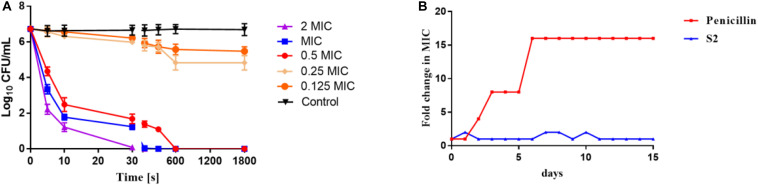
**(A)** Time-kill kinetic curves of S2 at 0.125 MIC, 0.25 MIC, 0.5 MIC, MIC, and 2 MIC against *S. aureus* 29213. **(B)** Emergence of bacterial resistance after treatment of *S. aureus* 29213 with antimicrobials for 15 days at sub-MIC concentration determined by sequential passaging method. The fold change in MIC of the antimicrobials was plotted against the number of days.

The time-dependent killing kinetics of S2 against *S. aureu*s was performed to ascertain whether there was an association between the time of exposure to AMPs and bacterial survival. As shown in Figure 1A, the concentration and time dependencies in the bacterial killing were observed. A 2 log10 bacterial reduction in *S. aureus* was observed at 0.5 × MIC after 5 s of antimicrobial exposure. S2 exhibited the faster sterilization rate at MIC and 2 × MIC concentrations, yielding 100% bacterial killing after only 60 and 30 s of peptide exposure, respectively.

### Evaluation of Bacterial Resistance

The development of antibiotic resistance should not be overlooked. *S. aureus* was serially treated in the presence of sub-MIC penicillin and S2 for 15 days, and the MIC value was tested every day. As shown in Figure 1B, the MIC value of penicillin was changed after two days, and the MIC value of the 15th day was 16-fold higher than the MIC value on the first day. However, the MIC value of S2 remained almost constant over the entire of 15 days.

### Circular Dichroism Spectra

**FIGURE 2:**
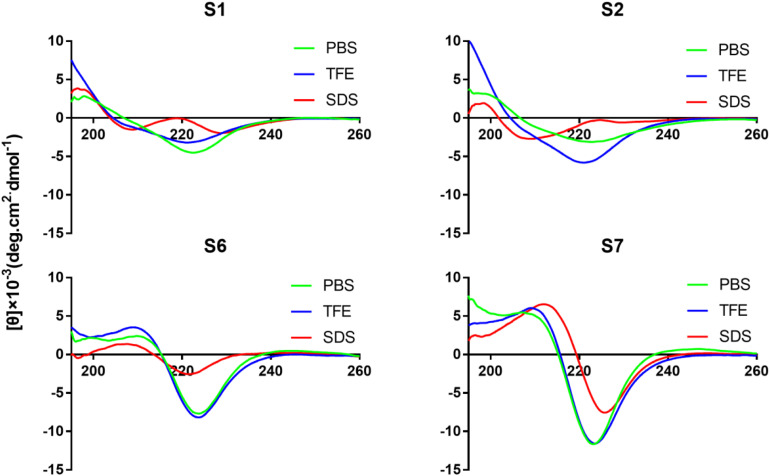
CD spectra of peptides (S1, S2, S6, and S7). All the peptides were dissolved in 10 mM PBS (pH 7.4), 50% TFE, or 30 mM SDS. The mean residual ellipticity was plotted against wavelength. The values from three scans were averaged per sample, and the peptide concentrations were fixed at 150 μM.

Circular dichroism spectroscopy was used to assay the secondary structures of peptides in different environments. Trifluoroethyl alcohol is known to induce helical structures in peptides and proteins, while SDS micelles are negatively charged and are used to mimic bacterial membranes. All Tryptophan-rich peptides exhibited a large negative band centered at 226 nm (Figure 2 and Supplementary Figure S1). S5 exhibited a negative peak at 208 nm and a weak positive peak at 228 nm, which indicated the formation of the turn conformation. The peptides without disulfide bonds (S6, S7, and S8) had a positive signal at 211 nm. S2 exhibited a negative peak at 208 nm, indicating that the targeting peptide tended toward an α-helical conformation in SDS.

### Peptide Interactions With LTA

**FIGURE 3:**
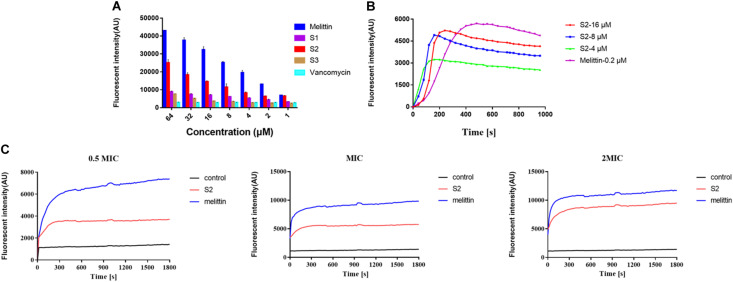
**(A)** LTA binding affinity of peptides and the antibiotic was determined using the BODIPY-TR cadaverine (BC) fluorescent dye displacement assay. The changes in fluorescence intensity were monitored at an excitation wavelength of 580 nm and an emission wavelength of 620 nm. The experiment was repeated three times independently. **(B)** Cytoplasmic membrane depolarization of *S. aureus* was monitored using membrane potential dye diSC_3_-5. **(C)** The fluorescence intensity of propidium iodide incubated with *S. aureus* 29213 treated by 1/2, 1, and 2 × MIC peptides were measured. Exponential-phase *S. aureus* cells were treated with melittin (blue), and S2 (red).

To investigate the ability of peptides to bind to LTA, a fluorescence-based displacement assay with BC was performed. The conventional anti-*S. aureus* vancomycin was used as the negative control because it inhibits cell wall synthesis in microorganisms. Melittin was chosen as the positive control. Figure 3A indicates that melittin, S1, S2, and S3 showed a concentration-dependent increase in fluorescence intensity, and S2 exhibited a stronger binding capacity to LTA than S1 and S3. In contrast, the fluorescence intensity induced by vancomycin was relatively weak, and as the concentration increased, the fluorescence intensity remained unchanged.

### Cytoplasmic Membrane Electrical Potential

The change in bacterial cytoplasmic membrane electrical potential caused by AMPs was determined using 3,3’-dipropylthiadicarbocyanine iodide (diSC_3_-5), a membrane potential-sensitive dye. The cytoplasmic membrane electrical potential was perturbed when S2 and melittin were added to the medium. The fluorescence intensity increased after diSC_3_-5 was released into the external environment. Figure 3B shows that the S2 and melittin act similarly, resulting in a rapid fluorescence intensity increase. Moreover, the fluorescence intensity increased with increasing S2 concentration.

### SEM and TEM Characterization

The SEM method was used to observe the *S. aureus* morphology and membrane integrity before and after cell incubation with S2. In contrast to the surface of the control bacteria, the surface of the treated bacterial cells with S2 at its MIC for 30 min showed wrinkles, disrupted membrane integrity, and leakage of cell contents, and the protoplasm of *S. aureus* showed viscidity (Supplementary Figure S2).

TEM showed changes in morphology and internal structure after treatment with S2 for 30 min. Supplementary Figure S2C shows that *S. aureus* has intact external structures and dense internal structures. In the presence of S2, the membrane of *S. aureus* was damaged, and a substantial outflow of cytoplasm was observed (Supplementary Figure S2).

### Propidium Iodide Uptake Assay

Propidium Iodide dye can enter cells after the membrane is disrupted, bind nucleic acids, and emit fluorescence. In the absence of peptide, cells showed almost no PI fluorescent signaling, indicating viable cell membranes. However, cells exhibited an increase in PI fluorescent signaling when S2 and melittin were added to the medium, respectively. Figure 3C revealed that, at the concentrations of 0.5 × MIC, MIC, and 2 × MIC, the changes in the fluorescence value depended on time and dosage. These results indicated that S2 and melittin rapidly disrupted the bacterial membrane and caused PI uptake.

### Cytotoxicity Measurements

**FIGURE 4:**
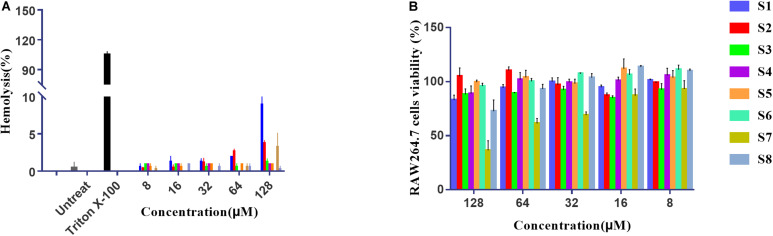
The hemolysis activity of peptides to human red cells **(A)**. Cytotoxicity of the peptides to RAW264.7 cells determined using the MTT method at different concentrations **(B)**.

The hemolytic activity of peptides was determined by causing low-grade 10% hemolysis against hRBCs at all concentrations of peptides. Figure 4A summarizes the peptide hemolytic activities. Compared with 0.1% Triton X-100, all AMPs had lower hemolytic activity. To further study the cell selectivity of these peptides, the cytotoxicity of peptides against RAW 264.7 cells was evaluated. Figure 4B shows that the RAW 264.7 cell survival rates of S1, S2, S3, S4, S5, S6, and S8 were 86, 105, 84, 90, 95, 94, and 85% at the highest concentration of 128 μM, respectively. However, higher concentrations of S7 had a negative effect on cell viability, with the lowest viability measured at almost 50%.

### Effect of Salts on Antimicrobial Activity

For practical applications, AMPs must maintain high activity under physiological conditions. Thus, the antimicrobial activity was determined in physiological concentrations of various salts (Table 4). For Ca^2+^ and Fe^3+^, the antimicrobial activity of S2 was reduced by half, which is within tolerable limits. Other cations did not affect the bacterial killing capacity of S2. Moreover, in the presence of Zn^2+^, not only was the MIC value of S2 was decreased, but the antimicrobial activity of S1 and S6 increased. Overall, all AMPs had a high tolerance to physiological salts.

**TABLE 4:** MIC values of the peptides against *S. aureus* 29213 in the presence of physiological salts.

**Peptides**	**MIC**	**NaCl^a^**	**KCl^a^**	**NH_4_Cl^a^**	**MgCl_2_^a^**	**CaCl_2_^a^**	**FeCl_3_^a^**	**ZnCl_2_^a^**
S1	4	4	4	4	4	8	8	1
S2	8	8	8	8	8	16	16	4
S3	>64	>64	>64	>64	>64	>64	>64	>64
S4	>64	>64	>64	>64	>64	>64	>64	>64
S5	>64	>64	>64	>64	>64	>64	>64	>64
S6	16	8	16	16	32	32	32	4
S7	8	8	8	8	8	16	8	4
S8	>64	>64	>64	>64	>64	>64	>64	64

*^*a*^The final concentrations of NaCl, KCl, NH_4_Cl, MgCl_2_, ZnCl_2_, CaCl_2_, and FeCl_3_ were 150 mM, 4.5 mM, 6 μM, 2 mM, 8 μM, 1 mM, and 4 μM, respectively, and the control MIC values were determined in the absence of these physiological salts. The data were derived from a representative value of three independent experimental trials.*

### *In vivo* Antibacterial Activity Assay

**FIGURE 5:**
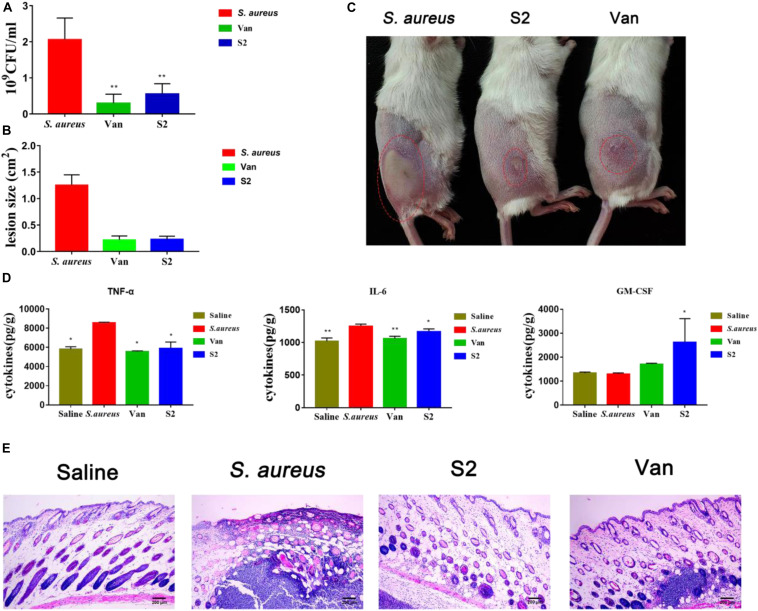
**(A)** Viable bacteria count at the infection site on day 2 post-infection. **P < 0.01; and *P < 0.05. *n* = 8. Data represents the mean ± SEM. Means with different letters represent significantly different at *P* < 0.05 by Duncan’s test. **(B)** lesions size. **(C)** Picture was taken at that time, the red circles indicate the infectious sites. From left to right, *S. aureus*, S2, vancomycin. **(D)** Effects of S2 and Van on cyst cytokines. **P < 0.01; and *P < 0.05. Data represents the mean ± SEM. Means with different letters represent significantly different at *P* < 0.05 by Duncan’s test. **(E)** HE photomicrograph of the skin tissue.

To study the therapeutic effects of the peptide, a mouse model of back skin infection was used to establish *in vivo* antibacterial activity. Intradermal *S. aureus* injections delivered the bacteria into the area between the epidermal and the subcutaneous layers of the skin. Saline, vancomycin, and S2 were respectively injected into the infected site after 1 h of infection. The infected tissues were homogenized in saline, diluted tissues were plated on MHA, and colonies were counted after 24 h (Figure 5A). Compared with the control group mice, the mice injected with vancomycin and S2 showed remarkable antimicrobial activity, which reached a 10-fold decrease in bacterial colonies (*P* < 0.01). We also examined the size of the dermatocyst to assess the contribution of S2 and vancomycin to reduce skin ulceration ability. The lesion size, equal to length (cm) × width (cm), was utilized to measure the extent of the damage. As shown in Figures 5B,C S2 and vancomycin significantly reduced ulceration sizes, and the lesion was relatively larger in the absence of treatment.

### Inhibition of Pro-inflammatory Cytokines and Stimulation of Anti-inflammatory Cytokines

The effect of S2 on the immune response induced by *S. aureus* was also measured. In this experiment, pro-inflammatory cytokines IL-6, TNF-α, and anti-inflammatory cytokines GM-CSF, were measured using an ELISA kit. As shown in Figure 5D, 24 h after treatment with Van and S2, pro-inflammatory cytokine (TNF-α and IL-6) generation was suppressed (*P* < 0.05). Granulocyte-macrophage colony-stimulating factor levels of S2-treated mice were significantly higher than those in the corresponding control group and Van group mice (*P* < 0.05). These results provided additional confirmation that S2 can effectively inhibit *S. aureus* subcutaneous infections.

### Histology of Skin Tissue

Histomorphological changes were measured to assess skin integrity (Figure 5E). There are intact skin structures, including epidermal, dermal, and subcutaneous adipose layers for healthy tissue. The *S. aureus* group showed inflammation, as represented by the excessive swelling of the dermal layer, and severe suppurative inflammatory infiltration was also observed. The skin tissues of S2 and Van groups displayed good recovery, which showed that inflammation in the dermal layer was reduced. In addition, the S2 group, which displayed minimal inflammation, was superior to the Van group.

### Toxicity of Peptides

**FIGURE 6:**
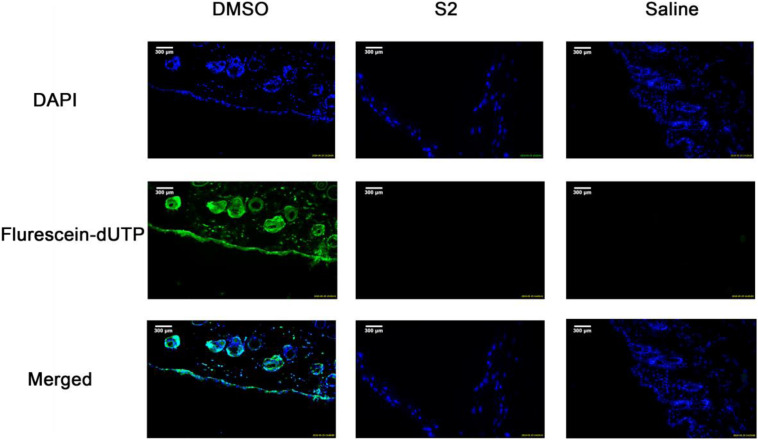
TUNEL assay of apoptotic cells in mouse skin. DMSO, Saline, and S2 were injected into the superficial dorsal skin of mice. 24 h post injection, the skin was collected for TUNEL assay and the nuclei of the cells were counterstained with DAPI, a blue fluorescent dye. The apoptotic cells were shown in green in the images.

To evaluate the effect of S2 on healthy skin tissues, ICR mice skin treated with S2, 100% DMSO, or saline were stained with fluorescein dUTP (fluorescein Deoxyuridine Triphosphate) for apoptotic cells and DAPI for cell nuclei. Apoptotic cells in the epidermal layer and dermal layer were stained green, and the nuclei were counterstained as blue. As shown in Figure 6, no apparent epidermal apoptosis was found in the S2 and saline groups. In contrast, the 100% DMSO group exhibited a thick epidermal layer of apoptotic cells; as previously reported, this treatment is toxic to skin. In addition, the quantification of apoptosis is shown in Supplementary Figure S3. To evaluate the safety of the peptide in *vivo*, we studied the toxicity of S2 injected intraperitoneally at 80 MIC with 100 μL. The treatment group of S2 performed well; there were no clinical signs indicative of toxicity (Supplementary Figure S4A). Compared with the saline treatment group, the blood parameters of the S2 treatment group were unaltered (Supplementary Table S1 and Supplementary Figures S4B,C). All these results indicated that 100 μL of S2 is unlikely to be toxic when treated at 80 MIC.

## Discussion

Leucine (L) reduces the energy required for the peptide to form a helix, which increases antimicrobial activity (Pace and Scholtz, 1998; Torres et al., 2018). Arginine (R) was used because its side chain guanidinium groups can form extensive and strong H-bonds to mediate the interaction of the anionic membrane of bacteria and peptides, which improves the lytic abilities of peptides (Zou et al., 2007). Therefore, S6 and S7 exhibited higher antimicrobial activity than S8. Compared with S4 and S7, the conjugation of the pheromone (without the thiolactone macrocycle) to the active peptide (S7) decreased the inherent antimicrobial activity of S7 because the inclusion of short peptides changes the structural parameters of the fusion peptide and affects the antimicrobial potency (Ma et al., 2015; Torres et al., 2019; Wang et al., 2019a). The addition of disulfide bonds to the targeting domain enhanced the antimicrobial selectivity against *S. aureus*, and the modified peptide S2 exhibited specific anti-*S. aureus* potency, suggesting that disulfide bonds play an important role in the targeting activity against *S. aureus* (Leeuw et al., 2007). Although S6 showed similar antimicrobial activity as S7, S1 exhibited broad-spectrum antimicrobial activity due to the high number of positive charges, resulting in a high accumulation on anionic bacterial membranes (Yang et al., 2019). S3 and S8 showed no antimicrobial activity due to the decrease in hydrophobicity, resulting in a reduction in the membrane destruction capacity (Schmidtchen et al., 2014). In contrast to S1 and S3, S2 showed a stronger targeting antimicrobial capacity against *S. aureus*, due to leucine, and less positive charges kill Gram-positive bacteria more effectively (Mishra and Wang, 2012; Wang et al., 2016). As Figure 1 shows, S2 exhibited a rapid sterilization effect, which was mainly dependent on the mechanism of action. In addition, because of the different mechanisms, it is more difficult to develop drug resistance to S2 compared to penicillin.

There have been some reports that conformational changes of AMPs occur in some solutions (Takahashi et al., 2010). Our results indicated that both S2 and S4 exhibited a large negative signal centered at 226 nm. Thus, in addition to the disulfide bond (Cys1–Cys6), the stacking of Trp also formed the structure of the turn (Ladokhin et al., 1999). S2 transformed into an α-helical structure in the membrane-mimetic environment, which could induce higher antimicrobial activity. There appears to be a positive signal at 211 nm and a negative signal at 225 nm in S6, S7, and S8, which indicated a splitting of the excitation states due to stacked Trp aromatic chromophores (Ladokhin et al., 1999). The curve of S5 intercepted the x-axis at approximately 226 nm, and the CD spectrum showed a negative band at 210 nm and a positive band at 229 nm, which could represent the turn structure, possibly combined with some parts of the unordered structure (Andrade et al., 1993).

The ability of AMPs to exert selective membrane destruction depends on the degree of enrichment near the bacteria. S2 exhibited stronger binding affinity to LTA than S1 and S3 due to the membrane carrier of LTA exhibiting hydrophobicity, which attracted the hydrophobic molecules of S2. The local peptide concentrations on the bacterial membrane increased, resulting in an enhanced membrane disruption capacity and antimicrobial activity. The fluorescence increase of diSC3-5 induced by S2 and melittin indicated cytoplasmic membrane depolarization. A previous study showed that the antimicrobial mechanism of AMPs might occur via the toroidal pore, barrel-stave, and carpet models or by electroporation and induced separation of membrane lipids (Li et al., 2012; Lv et al., 2014). The results of SEM and TEM demonstrated that S2 caused morphological changes in *S. aureus*, and the membranes of cells were disrupted, causing a leakage of its content. In addition, the internalization of the PI dye throughout the bacterial cytosol and the rapid increase in PI fluorescent signaling indicated that S2 killed bacterial cells by compromising the cell membrane integrity. However, whether targeting AMPs use similar antimicrobial mechanisms as the broad-spectrum AMPs to kill bacteria, including inhibition of cell wall biosynthesis (Wiedemann et al., 2001), pore formation in bacterial membranes, inhibition of spore outgrowth (Pag and Sahl, 2002), Lipid II-dependent pore formation (Farinas et al., 1999), and activation of autolytic enzymes (Bierbaum and Sahl, 1985), requires further study.

Natural AMPs nonspecifically target pathogenic microorganisms and cause membrane destruction in mammalian cells, which is one of the critical limits in the clinical application of AMPs. Hemolysis and cytotoxicity analysis revealed the biocompatibility of the peptides. Compared to S7, for which only 50% of the cells survived, S2 exhibited no killing activity against RAW 264.7 cells, suggesting that the active peptide linked with the targeting domain showed excellent cellular selectivity for zwitterionic mammalian and anionic bacterial membranes. Interestingly, unlike the cytotoxicity result, S7 and all the other peptides barely exhibited hemolysis effects. Therefore, we speculated that S7 might act on the nucleus or another organelle of mammalian cells but does not affect human erythrocytes that do not contain a nucleus (Lum et al., 2015; Wang et al., 2019b).

Cations decrease the electrostatic effect between the positive charge of peptides and the negative charge on the surface of bacteria, leading to reduced antimicrobial activity (Huang et al., 2011b). In the presence of Ca^2+^ or Fe^3+^, the antimicrobial activity of S2 was compromised, which may be due to competition between divalent or trivalent ions and AMPs. Studies have shown that the addition of Ca^2+^ and Mg^2+^ to the medium results in an 80% loss of bacterial killing efficacy (Qian et al., 2018). However, the MIC value for S2 was doubled in the presence of Ca^2+^ in the assay medium. It has been proposed that the addition of a relatively small number of divalent cations could be helpful for the binding of AMPs to bacterial membranes (Dong et al., 2014), S2 shows a synergistic effect with Zn^2+^, resulting in increased activity.

Subcutaneous infections are the most common form of *S. aureus*, causing approximately 70% of osteomyelitis and 80% of joint infections in patients with rheumatoid arthritis and several other bone diseases (Dusane et al., 2018). In our study, S2 significantly reduced bacterial counts and dermatocyst size, which was similar to the effect of vancomycin. In addition to reducing the bioburden in wound tissue, AMPs can modulate the immune response by stimulating leukocyte recruitment to the site of infection and the production of immune mediators GM-CSF (Torres and de la Fuente-Nunez, 2019), and inhibit *S. aureus*-evoked expression of the pro-inflammatory cytokines of TNF-α and IL-6. TNF-α and IL-6 are the primary mediators of sepsis, which can cause septic shock(Li et al., 2017). Granulocyte-macrophage colony-stimulating factor is used clinically to stimulate hematopoiesis following chemotherapy and promote the generation of white blood cells (Zhan et al., 2012). Meanwhile, *S. aureus* promotes the development of T helper (Th) cells (Jiang et al., 2017). Activated B cells, T cells, and the Th17 response can induce GM-CSF, which leads to the mobilization of macrophages, neutrophils, eosinophils, and dendritic cell maturation (Fleetwood et al., 2005). The results of TNF-α, IL-6, and GM-CSF indicated that S2 could effectively modulate the immune response. In addition, the safety studies in *vivo* indicated that S2 have no adverse effect on healthy mice, which further suggests that it is a promising candidate for the treatment of *S. aureus* infections.

In this study we successfully synthesized the targeting AMP S2 by linking the targeting domain of the modified quorum-sensing signal pheromone to the killing domain. The mechanism of action studies indicated that S2 killed bacteria by physically disrupting the membrane, causing intracellular content leakage. In addition, the design of AMPs in this study showed high biocompatibility and salt stability. Mouse model studies demonstrated that S2 still had potent antimicrobial activity *in vivo*, and reduced bioburden and promoted wound healing. Overall, this study successfully designed a targeting AMP against *S. aureus*, and laid the foundation for the development of targeting AMPs.

## Data Availability Statement

All datasets generated for this study are included in the article/Supplementary Material.

## Ethics Statement

The studies involving human participants were reviewed and approved by Northeast Agricultural University. The patients/participants provided their written informed consent to participate in this study. The animal study was reviewed and approved by Northeast Agricultural University.

## Author Contributions

LS and JL carried out the conception and design and the acquisition of data. CS conducted the mice experiments. ZN, QL, SC, and ZW conducted the SEM and TEM assay. AS designed and conceived the experiments. All authors contributed to the article and approved the submitted version.

## Conflict of Interest

The authors declare that the research was conducted in the absence of any commercial or financial relationships that could be construed as a potential conflict of interest.
