# Supplementary material for: Hybrid Antimicrobial Peptide Targeting Staphylococcus aureus and Displaying Anti-infective Activity in a Murine Model
Source: Front Microbiol. 2020 Sep 11;11:1767. doi: 10.3389/fmicb.2020.01767 (PMC7516806; doi:10.3389/fmicb.2020.01767)
Supplement: Supplementary file 1 [file Data_Sheet_1.PDF]

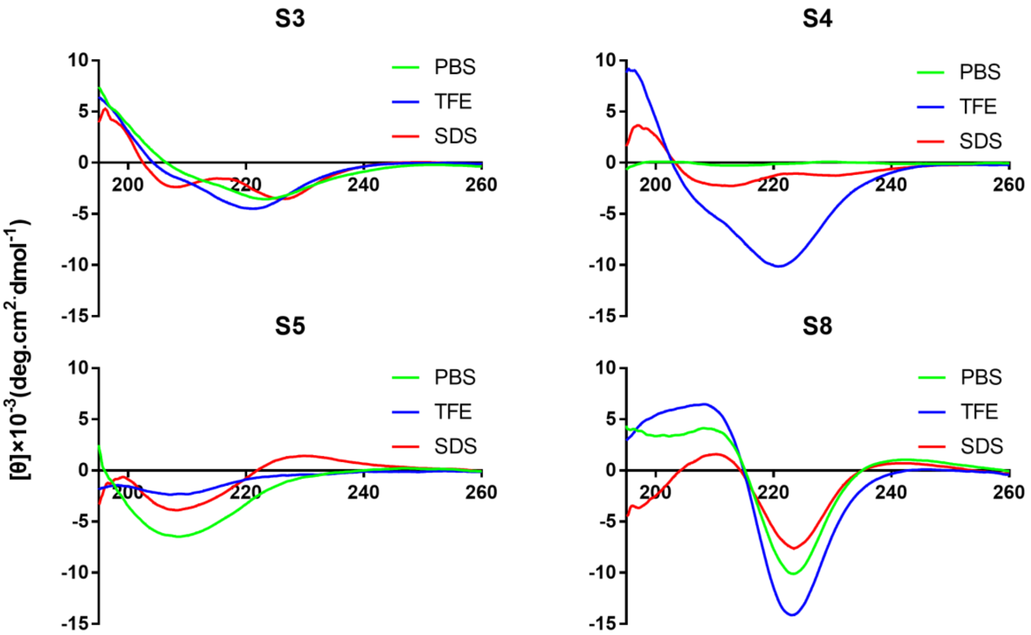

Figure S1. CD spectra of peptides (S3, S4, S5 and S8). All the peptides were dissolved in 10 mM PBS (pH 7.4), 50% TFE, or 30 mM SDS. The mean residual ellipticity was plotted against wavelength. The values from three scans were averaged per sample, and the peptide concentrations were fixed at 150 μM.

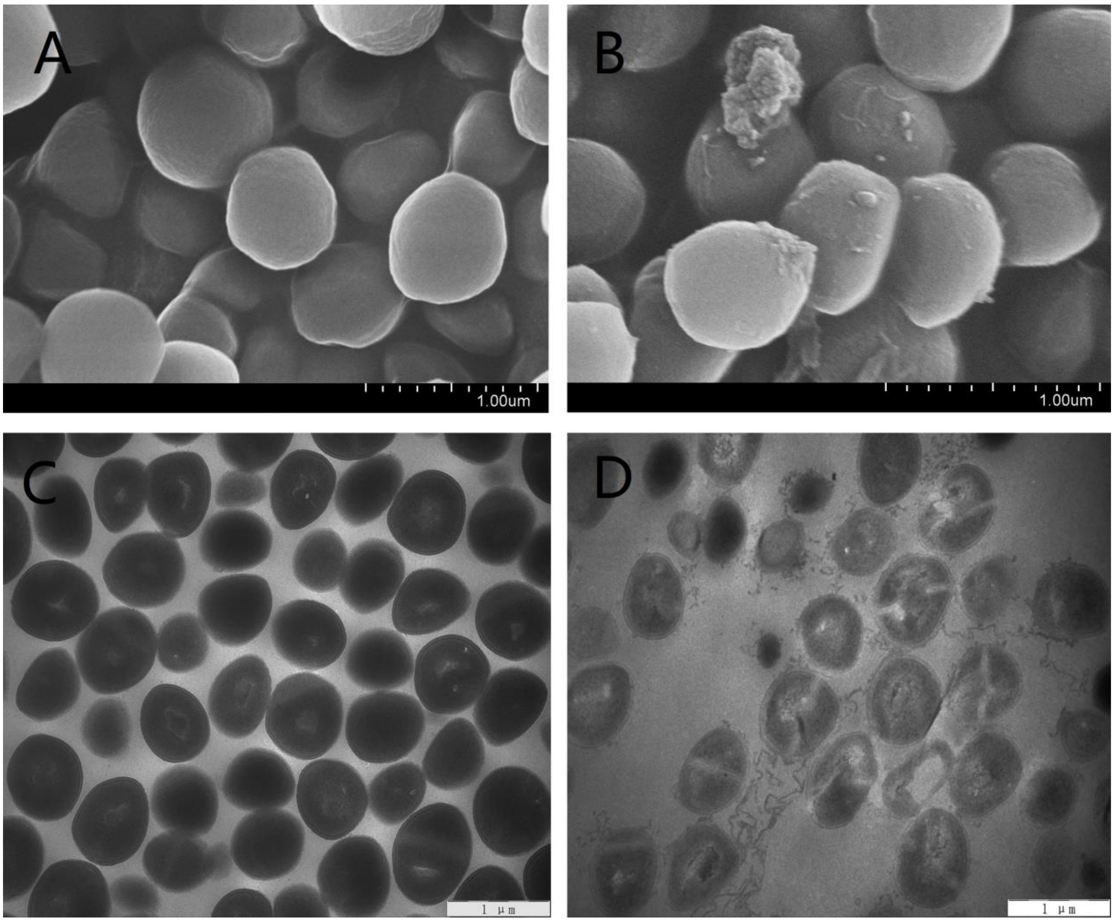

Figure S2. Membrane damage effect of *S. aureus* 29213 treated by S2 was observed by SEM (B) and TEM (D). (A, C) Control， no peptides.

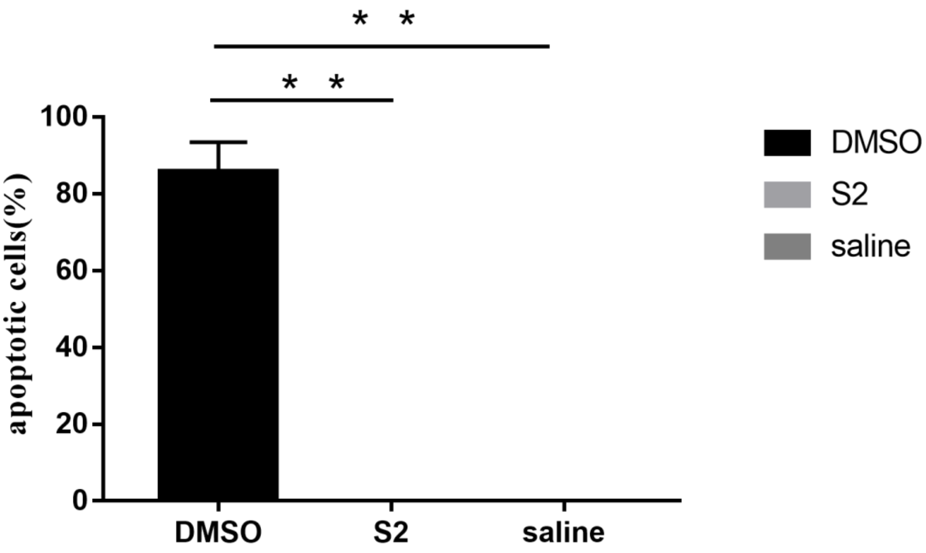

Figure S3. Quantification of apoptosis cells using the TUNEL stained images with Image J software. Values are mean ± SEM. ** $P < 0.01$.

Table S1 The effect of S2 on blood biochemistry parameters

	Na^+ mmol/L	K^+ mmol/L	Cl^- mmol/L	I.Phos. mmol/L	Glucose mmol/L	Urea mmol/L	Creatinine mmol/L
S2							
Mean	149	5.84	111.25	2.335	10.17	9.43	12.4
SD	0.926	0.562	1.035	0.373	0.529	0.459	1.6
Saline							
Mean	147.8725	5.7225	112.37	2.335	10.166	9.537	12.16
SD	2.167	0.475	2.13	0.3733	0.53	0.46	1.60

Normal values are 145–160 mmol/L for Na+, 4–7.5 mmol/L for K+, 110–120 mmol/L for Cl−, 1.2–2.8 mmol/L for inorganic phosphorus, 9.5–11 mmol/L for glucose, 9–12 mmol/L for urea, and 6–14 μmol/L for creatinine.

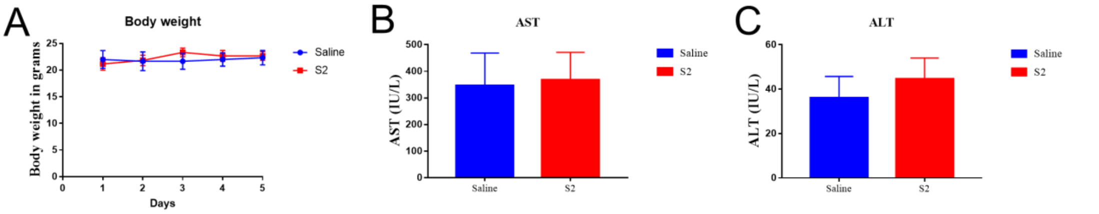

Figure S4. (A) Change in body weight of mice over time upon administration of peptide S2 monitored daily for five days. (B) Concentrations of aspartate transaminase (AST) for each mouse serum sample. (C) Concentration of alanine aminotransferase (ALT) for each mouse serum sample.
